# Clinical Utility of the Prenatal BACs-on-Beads™ Assay in Invasive Prenatal Diagnosis

**DOI:** 10.3389/fgene.2021.789625

**Published:** 2022-01-14

**Authors:** Yu Jiang, Lili Wu, Yunshen Ge, Jian Zhang, Yanru Huang, Qichang Wu, Yanhong Zhang, Yulin Zhou

**Affiliations:** ^1^ United Diagnostic and Research Center for Clinical Genetics, Women and Children’s Hospital, School of Medicine and School of Public Health, Xiamen University, Xiamen, China; ^2^ Department of Obstetrics and Gynecology, Women and Children’s Hospital, School of Medicine, Xiamen University, Xiamen, China; ^3^ Department of Medical Ultrasonics, Women and Children’s Hospital, School of Medicine, Xiamen University, Xiamen, China

**Keywords:** prenatal bacs-on-beads™, clinical utility, prenatal diagnosis, applicable populations, clinical pathways

## Abstract

**Background:** The prenatal BACs-on-Beads™ (PNBoBs™) assay has been applied worldwide for prenatal diagnosis. However, there are neither guidelines nor consensus on choosing patients, sample types, or clinical pathways for using this technique. Moreover, different perspectives have emerged regarding its clinical value. This study aimed to evaluate its clinical utility in the context of clinical practice located in a prenatal diagnostic center in Xiamen, a city in southeast China.

**Methods:** We tested 2,368 prenatal samples with multiple referral indications using both conventional karyotyping and PNBoBs™. Positive results from PNBoBs™ were verified using current gold-standard approaches.

**Results:** The overall rates for the detection of pathogenic copy number variation (pCNV) by karyotyping and PNBoBs™ were 1.9% (46/2,368) and 2.0% (48/2,368), respectively. The overall detection rate of karyotyping combined with PNBoBs™ for pCNV was 2.3% (54/2,368). A total of 13 cases of copy number variation (CNV)with a normal karyotype were detected by PNBoBs™. Another case with a normal karyotype that was detected as a CNV of sex chromosomes by PNBoBs™ was validated to be maternal cell contamination by short tandem repeat analysis.

**Conclusion:** Karyotyping combined with PNBoBs™ can improve both the yield and efficiency of prenatal diagnosis and is appropriate in the second trimester in all patients without fetal ultrasound anomalies who undergo invasive prenatal diagnosis.

## Introduction

Since its invention (Gross et al., 2011), PNBoBs™ has been rapidly adopted worldwide and has proven to be a reliable and rapid molecular cytogenetic technique for the detection of common aneuploidies (Trisomy or monosomy of chromosomes 13, 18, 21, X and Y) and frequently-occurring microdeletion syndromes (Wolf-Hirschhorn, Cri du Chat, Williams-Beuren, Langer-Giedion, Prader-Willi/Angelman, Miller-Dieker, Smith-Magenis, and Di-George) in invasive prenatal diagnosis (Vialard et al., 2012; Łaczmańska and Stembalska, 2013; Choy et al., 2014; García-Herrero et al., 2014; Rosenfeld et al., 2014; Grati et al., 2015; Li et al., 2018; Miao et al., 2019). However, to date, there is still no consensus on which patients undergoing invasive prenatal diagnosis are appropriate for PNBoBs™ testing and how to use its results to make clinical decisions. However, with the use of genome-wide copy-number variation detection techniques, including chromosome microarray analysis (CMA) and copy number variation sequencing (CNV-seq) in prenatal diagnosis (Stosic et al., 2018; Wang H. et al., 2020), doubts have been raised regarding the clinical utility of PNBoBs™ in invasive prenatal diagnosis (Xu et al., 2020). In this article, we present our recommendations on these issues based on our analysis of laboratory findings from one of the largest prenatal diagnosis centers in southeastern China.

## Materials and Methods

### Study Participants

A total of 2,368 prenatal diagnostic samples were collected at the Women and Children’s Hospital, School of Medicine, Xiamen University (Xiamen, China) between January 2018 and August 2021. Most (2,364) were amniotic fluid; the remaining four were umbilical cord blood. The indications for prenatal diagnosis included advanced maternal age (AMA, ≥35 years), high-risk maternal serological screening (HR-MSS), high-risk noninvasive prenatal testing (HR-NIPT), fetal ultrasound soft marker abnormality (FUSMA), fetal ultrasound structural abnormality (FUSA), adverse pregnancy history (APH), and other indications, such as carriers of single-gene disorders/chromosomal polymorphisms, history of exposure to radiation/drug during pregnancy, and assisted reproduction. Ethical approval was obtained from the Ethical Review Committee of the Women and Children’s Hospital, School of Medicine, Xiamen University (No. KY-2017-058). All patients provided written informed consent before participation.

### Karyotyping

G-banded karyotyping was performed using standard procedures at the 320–400 band level. Fetal karyotypes were classified according to the International System for Human Cytogenetic Nomenclature 2016 (ISCN 2016). Karyotyping was used as the gold standard for numerical chromosomal abnormalities, balanced chromosomal structural rearrangements, and mosaicisms.

### PNBoBs™

DNA was extracted from approximately 10 ml of amniotic fluid or 0.2 ml of umbilical cord blood using the QIAamp DNA Mini Kit (cat. no. 51306; Qiagen, Inc.), according to the manufacturer’s protocol. DNA concentration and purity were assessed using a NanoDrop One spectrophotometer (Thermo Fisher Scientific, Inc.,). The PNBoBs™ (cat. no. 3100-0020; PerkinElmer, Inc.) assay was performed according to the manufacturer’s instructions, which state that the assay can detect aneuploidies in chromosomes 13, 18, 21, X, and Y, as well as nine common microdeletion syndromes. The fluorescence of sample DNA bound to the beads was measured using a Luminex® 200™ instrument system (Luminex Corporation). Raw data generated by the instrument were analyzed using BoBsoft™ software (version 1.1; PerkinElmer, Inc.). Results were interpreted using the optimization method described in our previous report (Jiang et al., 2021).

### Chromosome Microarrays

Cases classified as CNV in microdeletion syndrome regions, as well as partial loss/gain of the X chromosome by the PNBoBs™ assay were verified by CMA using the CytoScan® 750 K Array Suite kit (Cat. No. 901859; Affymetrix, Inc., Thermo Fisher Scientific, Inc.) according to the manufacturer’s protocol. Genomic data were analyzed using Chromosome Analysis Suite 4.0 (r28959) software (Affymetrix, Inc.; Thermo Fisher Scientific, Inc.). Meanwhile, the CMA was also used as the gold standard for verifying unbalanced chromosomal structural rearrangements and marker chromosomes detected by karyotyping. Variant pathogenicity was interpreted according to the American College of Medical Genetics and Genomics (ACMG) standards and guidelines (Riggs et al., 2020).

### Statistical Methods

Cohen’s κ coefficient was calculated to measure the concordance of the results between karyotyping and PNBoBs™.

## Results

### Detection Performance Stratified by Primary Indication for Prenatal Diagnostic

The 2,368 cases were categorized into seven subgroups by primary clinical indications for prenatal diagnosis. The numbers of abnormal and pathogenic results for each group are shown in Table 1. Supplementary Table S1 provides further details on the pCNV cases. The Cohen’s κ coefficient between the two methods (PNBoBs™ and karyotyping) was 0.85, with a concordance of 99.4%. The pCNV detection rate for PNBoBs™ was higher than that of karyotyping in the AMA and FUSA groups, but was lower in the HR-MSS group. The overall detection rate of karyotyping combined with PNBoBs™ for pCNVs was 2.3% (54/2,368).

**TABLE 1:** Detection performance of karyotyping versus PNBobs™ stratified by primary indication for prenatal diagnosis.

	Cases, n	Karyotyping results, n (%)		PNBobs™ results, n (%)	
Primary indication		Abnormal	Pathogenic	Abnormal	Pathogenic
AMA	815	17 (2.1)	4 (0.5)	7 (0.9)	5 (0.6)
HR-MSS	1,221	38 (3.1)	19 (1.6)	21 (1.7)	17 (1.4)
HR-NIPT	19	11 (57.9)	10 (52.6)	10 (52.6)	10 (52.6)
FUSMA^†^	46	6 (13.0)	5 (10.9)	5 (10.9)	5 (10.9)
FUSA	12	7 (58.3)	7 (58.3)	10 (83.3)	10 (83.3)
APH	104	3 (2.9)	1 (1.0)	1 (1.0)	1 (1.0)
Others^‡^	151	2 (1.3)	0 (0)	0 (0)	0 (0)
Total	2,368	84 (3.5)	46 (1.9)	54 (2.3)	48 (2.0)
Combined DR of pCNV	2.3% (54/2,368)				

a Including absent/hypoplastic nasal bone; choroid plexus cysts; echogenic bowel; echogenic intracardiac focus; intrauterine growth restriction; pyelectasis; aberrant right subclavian artery.

b Including carrier of single-gene disorders/chromosome polymorphisms; exposure history to radiation/drug/chemicals, etc. during pregnancy; assisted reproductive patients; pregnant complicated with congenital heart diseases; volunteered to undergo prenatal diagnosis.

Abbreviation: PNBoBs™: prenatal bacterial artificial chromosome (BACs)-on beads; AMA, advanced maternal age; HR-MSS, high risk of maternal serological screening; HR-NIPT, high risk of noninvasive prenatal testing; FUSMA, fetal ultrasound soft markers abnormality; FUSA, fetal ultrasound structural abnormality; APH, adverse pregnancy history; DR, detection rate; pCNV, pathogenic copy number variation.

### Common Aneuploidies and Supernumerary Marker Chromosomes

**FIGURE 1:**
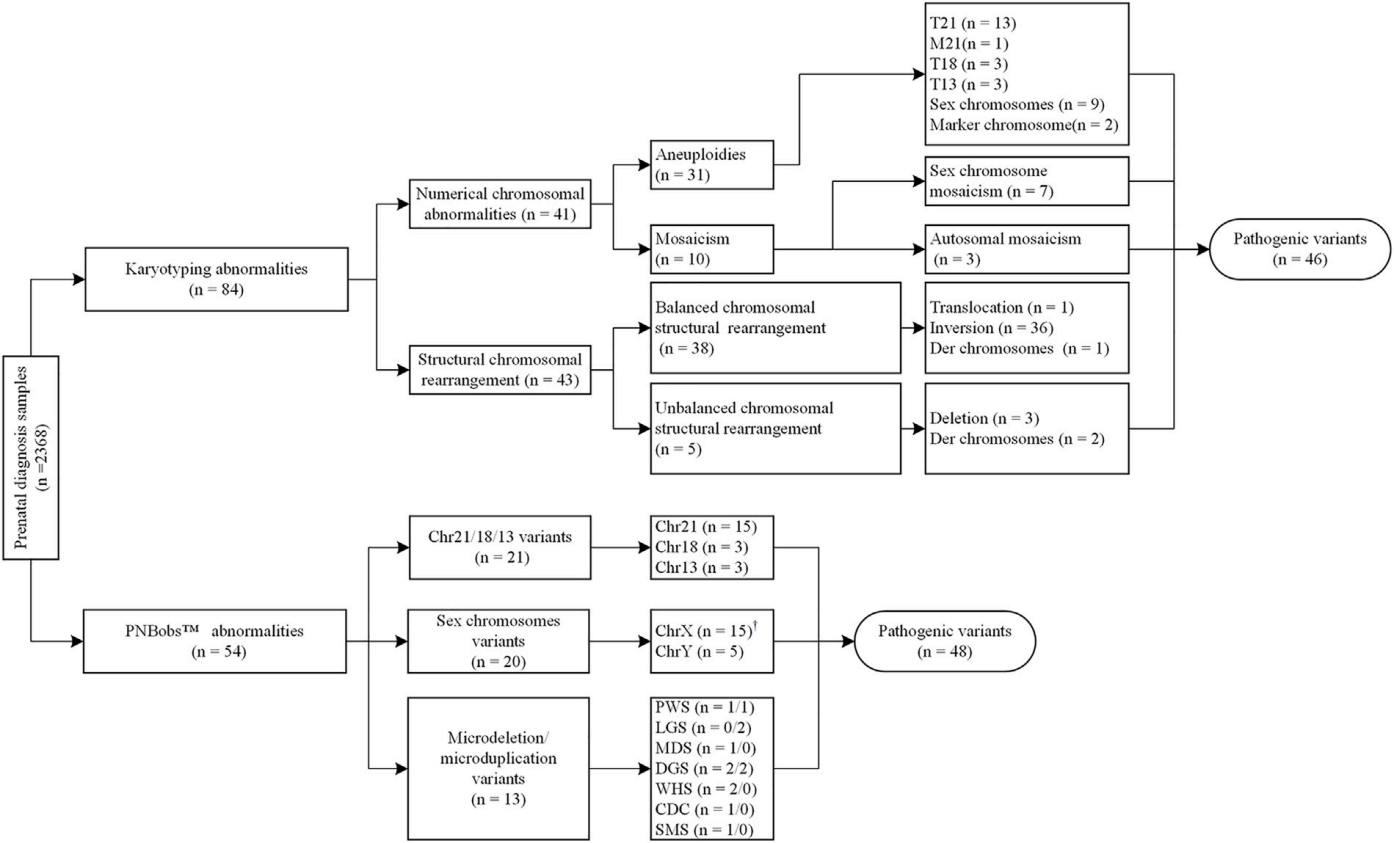
Overall results of karyotyping and prenatal bacterial artificial chromosome (BACs)-on beads (PNBoBs™) assay in 2,368 prenatal diagnosis cases.

As shown in Figure 1, 31 cases of chromosomal aneuploidy were detected by karyotyping, including 20 cases of autosomal aneuploidy, nine cases of sex chromosome aneuploidy, and two cases with supernumerary marker chromosomes. One case with supernumerary marker chromosomes was confirmed as a 6p11.2q14.1 duplication [arr (hg19) 6p11.2q14.1(57,590,402–77,659,343) × 3] using CMA; the other case was negative in confirmatory experiments. The PNBoBs™ assay successfully detected all 29 cases of common aneuploidies but missed the two cases of supernumerary marker chromosomes.

### Mosaic and Maternal Cell Contamination

**FIGURE 2:**
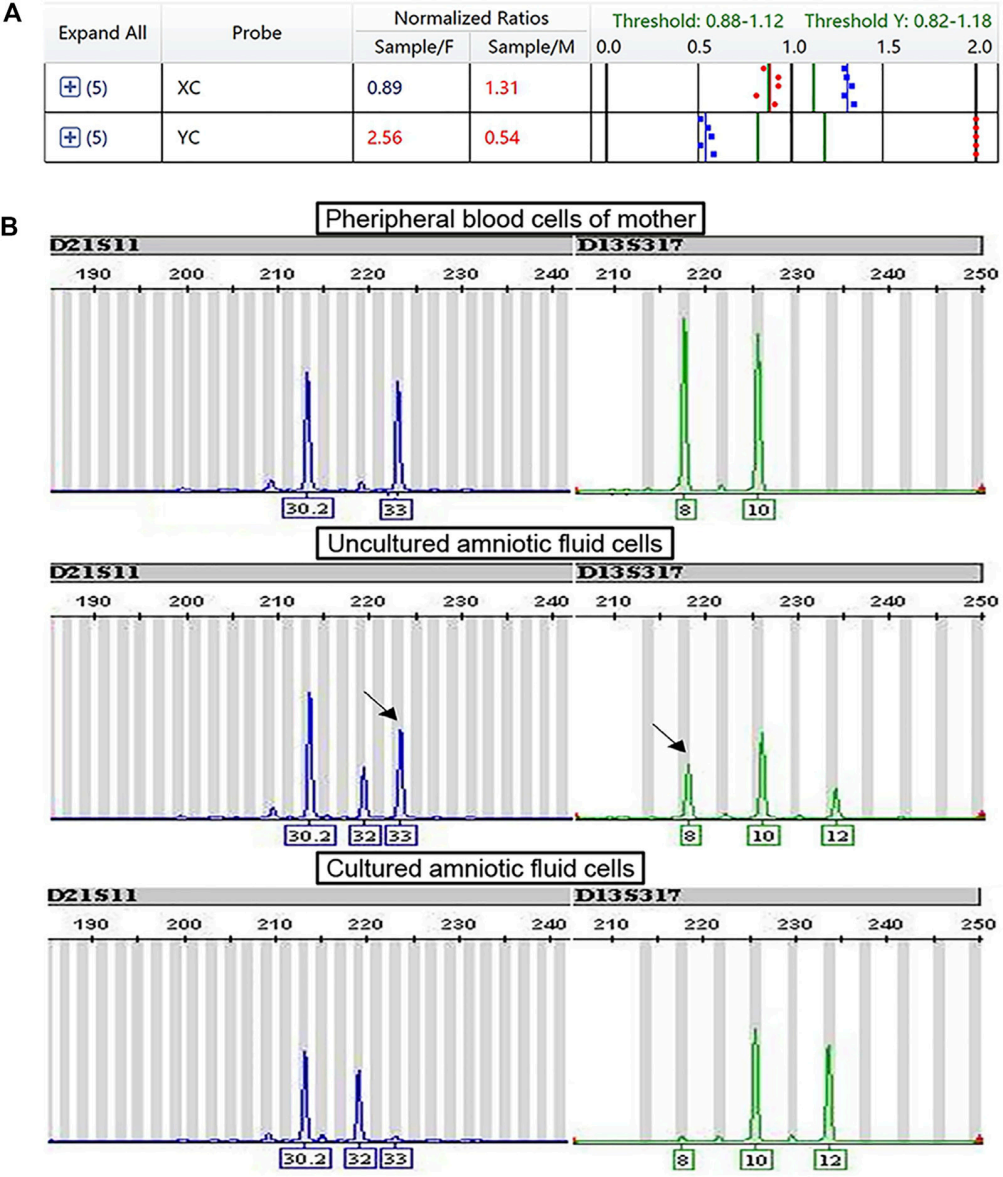
Profile of prenatal BACs-on-Beads™ and short tandem repeat analysis for the case with maternal cell contamination. **(A)** The mean normalized ratio and profile of sex chromosome of case B180010 against the references. The numbers in red indicate that the value exceeds the threshold. The numbers in blue indicate that the value is within the range of the threshold. **(B)** Profile of short tandem repeat analysis of peripheral blood cells of the mother, fetal uncultured amniotic fluid cells, and fetal cultured amniotic fluid cells (From top to bottom). The black arrow indicated the maternal contamination allele.

Ten cases of mosaicism were identified by karyotyping (Table 2). Of these, seven were sex-chromosome mosaicisms, and the remaining three were autosomal. All seven cases of sex-chromosome mosaicism were accurately detected by the PNBoBs™ assay, while all three cases of autosomal mosaicism were missed. One sample of amniotic fluid that was detected as a sex chromosome CNV using the PNBoBs™ assay had a normal male karyotype after cell culture; short tandem repeat analysis confirmed that the sample had been contaminated by maternal cells (Figure 2).

**TABLE 2:** Mosaicism and maternal cell contamination cases detected by karyotyping and/or PNBobs™.

case Id	Karyotype	Level of mosaicism	PNBobs™ results
B180010	46,XY	—	Detected
B180172	mos 45,X (63)/47,XXX (23)	73.3%/26.7%	Detected
B180088	mos 48,XX,+5,+12 (4)/46,XX (57)	6.6%	Undetected
B180347	mos 46,XY,r (22) (p11.2q13) (21)/46,XY (83)	20.2%	Undetected
B180458	mos 45,X (6)/46,XY (69)	8.0%	Detected
B180666	mos 45,X (8)/46,X, psu dic(Y) (q12) (52)	100%	Detected
B181267	mos 46,XX,t (3; 7) (q24; q11.2) (14)/46,XX (36)	28.0%	Undetected
B181588	mos 46,XY (45)/47,XXY (8)	15.1%	Detected
B190006	mos 45,X (37)/46,XX (20)	64.9%	Detected
B190019	mos 45,X (54)/46,XY (7)	88.5%	Detected
B190157	mos 45,X (19)/46,XX (31)	38.0%	Detected

Abbreviation: PNBoBs™: prenatal bacterial artificial chromosome (BACs)-on beads.

### Chromosomal Structural Abnormalities

Forty-three cases of structural abnormalities were detected by karyotyping, including 38 cases of balanced and five cases of unbalanced structural rearrangements (Figure 1). All fetuses with balanced structural rearrangements showed normal growth and development during subsequent follow-up and were delivered normally. Of the five cases with unbalanced structural variations, three deletions (two Wolf-Hirschhorn syndrome and one Cri du Chat syndrome) were detected using the PNBoBs™ assay within 48 h after sampling. Importantly, eight microdeletions and five microduplications detected by PNBoBs™ had normal karyotypes (Table 3 for details). After verification by CMA, all microdeletions were interpreted as pathogenic variations according to the ACMG guidelines (Riggs et al., 2020). Fetuses with autosomal microduplications had no abnormal ultrasound findings in subsequent follow-up, and their deliveries were uneventful.

**TABLE 3:** Details of microdeletion/microduplication cases detected by PNBobs™ assay.

case No.	Karyotype	Referral reasons	WG	Sample type	CMA result (range of variation)	PNBobs™ result	Parental origin	Pregnancy outcomes
1	46,XY	High risk with maternal serological screening	21^+2^	AFC	arr (hg19) 15q11.2q12 (23,651,596–27,214,745) × 3, 3.6 Mb	Dup PWS	unknown	NP
						C1-7		
2	46,XY	Intrauterine growth restriction	18^+2^	AFC	arr (hg19) 15q11.2q12 (22,770,421–27,199,122) × 1 dn, 4.4 Mb	Del PWS	*de novo*	TOP
						C1-7		
3	46,XX	Advanced maternal age	20^+2^	AFC	arr (hg19) 8q23.3 (116,143,860–117,081,995) × 3 mat, 938.1 Kb	Dup LGS	mat	NP
						C1-3		
4	46,XY	High risk with maternal serological screening	18^+1^	AFC	arr (hg19) 8q23.3 (118,443,810–118,981,861) × 3, 538.1 Kb	Dup LGS	unknown	NP
						C5-6		
5	46,XY	Severe ventriculomegaly; polyhydramnios	29^+1^	UCB	arr (hg19) 17p13.3 (525–2,815,682) × l dn, 2.8 Mb	Del MDS	*de novo*	TOP
						C1-6		
6	46,XX	High risk with maternal serological screening	17^+5^	AFC	arr (hg19) 22q11.21 (18,919,477–21,800,471) × 3 pat, 2.9 Mb	Dup DGS	pat	NP
						C1-4		
7	46,XY	High risk with maternal serological screening	19	AFC	arr (hg19) 22q11.21 (18,920,408–21,800,471) × 3, 2.9 Mb	Dup DGS	unknown	NP
						C1-4		
8	46,XY	Open spina bifida	24^+4^	UCB	arr (hg19) 22q11.21 (18,648,855–21,800,471) × 1 dn, 3.2 Mb	Del DGS	*de novo*	TOP
						C1-4		
9	46,XY	Intracardiac echogenic focus; thymic hypoplasia	27^+3^	UCB	arr (hg19) 22q11.21 (18,648,855–21,800,471) × 1 dn, 3.2 Mb	Del DGS	*de novo*	TOP
						C1-4		
10	46,XY	Advanced maternal age	19^+1^	AFC	arr (hg19) 17p11.2 (16,761,814–18,304,116)× 1 dn, 1.5 Mb	Del SMS	*de novo*	TOP
						C1-2		
11	46,XY	High risk with maternal serological screening	19	AFC	arr (hg19) Xp22.31 (6,455,151–8,141,076) × 0, 1.7 Mb	Del XC1	unknown	NP
12	46,XY	High risk with maternal serological screening	20^+5^	AFC	arr (hg19) Xp22.31 (6,455,151–8,141,076) × 0, 1.7 Mb	Del XC1	unknown	NP
13	46,XX	High risk with maternal serological screening	16^+2^	AFC	arr (hg19) Xp22.31 (6,455,151–8,143,509) × 1, 1.7 Mb	Del XC1	unknown	NP

Abbreviation: PNBoBs™: prenatal bacterial artificial chromosome (BACs)-on beads; PWS, Prader-Willi syndrome; LGS, Langer-Giedion syndrome; MDS, Miller-Dieker syndrome; DGS, DiGeorge syndrome; SMS, Smith-Magenis syndrome; TOP, termination of pregnancy; NP, normal pregnancy; pat, paternally inherited; mat, maternally inherited; AFC, amniotic fluid cells; UCB, umbilical cord blood; WG, weeks of gestation; Dup, duplication; Del, deletion; X, X-chromosome; C, clone.

### Clinical Recommendation for the Use of PNBoBs™

**FIGURE 3:**
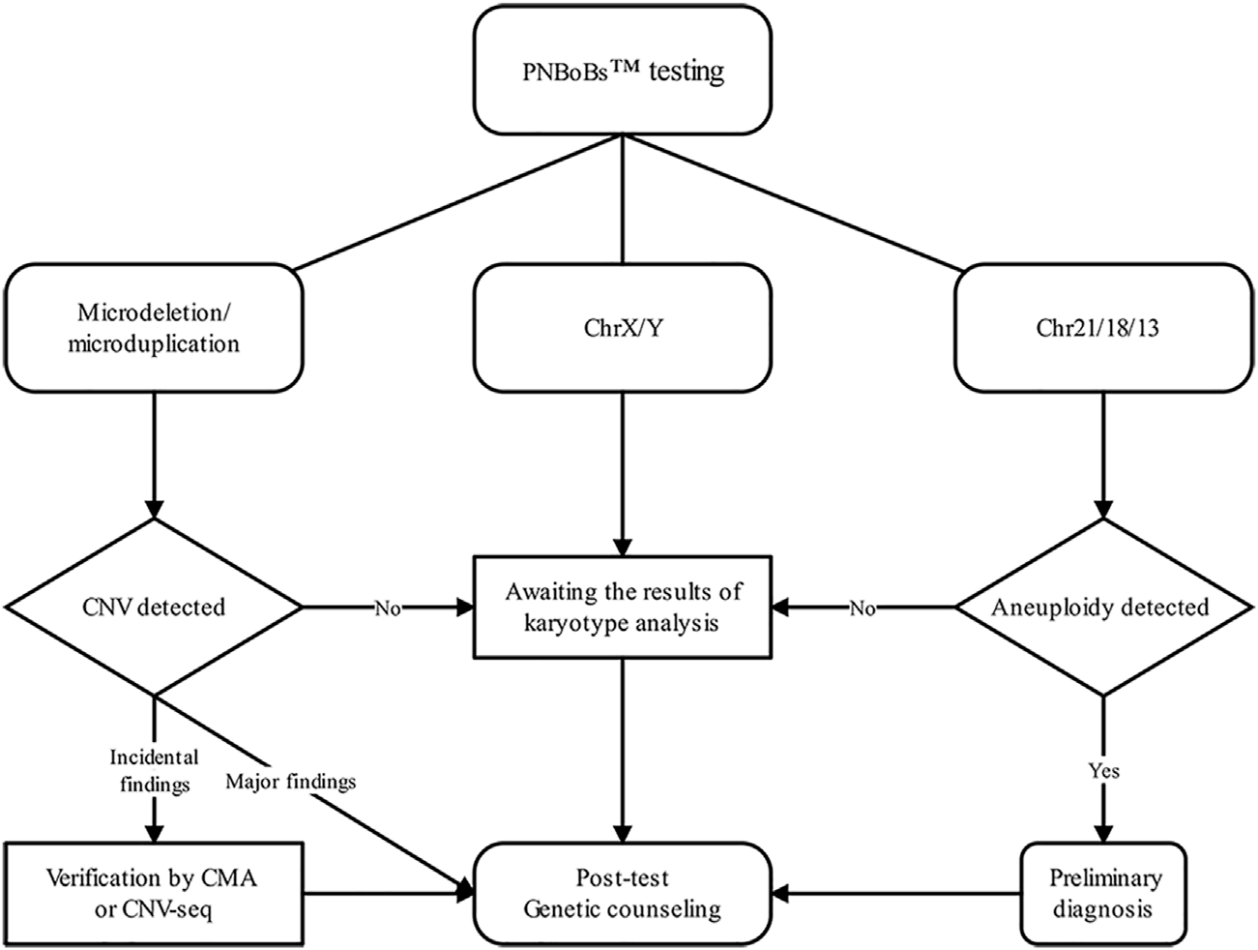
Flow diagram of the clinical pathway in prenatal diagnosis when combing using PNBoBs™ and karyotyping.

Based on these data and practical experience gained in this study, we established a recommended workflow for the clinical application of PNBoBs™ (Figure 3). Our primary recommendation is that when the mean normalized ratio of chr21/18/13 in the sample is within the 95% confidence interval of the reference established in the laboratory, the results can be used as the basis for the diagnosis of aneuploidy. Due to the relatively high prevalence of mosaicism, sex chromosome aneuploidies detected by PNBoBs™ are recommended in conjunction with karyotype results to determine whether the mosaicism is present or not. CNVs are classified as major findings when all the PNBoBs™ probe signals are missing in specific microdeletion syndrome regions. Such results are clinically relevant and can be used for clinical decision-making. Duplication/partial deletion in the microdeletion syndrome regions or X chromosome are classified as incidental findings. Due to the lack of sufficient validation data, at this stage, we propose verifying incidental findings by CMA/CNV-seq to assess the pathogenicity of these CNVs. For the cases with negative results by both karyotyping and PNBoBs™, the limitations of the methodology and residual risk should be discussed in post-test genetic counseling, as well as the clinical recommendations when additional fetal anomalies are identified in the subsequent follow-ups.

## Discussion

Previous studies revealed that common aneuploidies and recurrent pathogenic copy number variations are the main etiologies of birth defects (Grati et al., 2015; Feldkamp et al., 2017). Therefore, to prevent birth defects, it is critical to confirm these abnormalities in fetuses during pregnancy. Currently, a definitive prenatal diagnosis can only be made through invasive testing to obtain fetal tissue or cells for genetic analyses. In many countries, karyotyping is a criterion test for all patients undergoing prenatal diagnosis and is regarded as the gold standard for numerical chromosomal abnormalities, mosaicisms, and structural rearrangements. However, karyotyping has several limitations: it is expensive, labor-intensive, low-throughput, and has limited ability to detect minor structural rearrangements (Farra et al., 2020). Therefore, the question remains open: What additional assays should be offered to patients that can detect pathogenic variations in a high-throughput, accurate, and timely manner?

In 2016, the American College of Obstetricians and Gynecologists (ACOG) and the Society for Maternal-Fetal Medicine (SMFM) recommended CMA for prenatal diagnosis of cases with one or more fetal structural abnormalities (Vora et al., 2016). In 2018, the Society of Obstetricians and Gynecologists of Canada (SOGC) and the Canadian College of Medical Geneticists (CCMG) recommended that rapid aneuploidy detection (RAD) be performed prior to CMA when multiple fetal malformations are detected or nuchal translucency (NT) is ≥ 3.5 mm (Armour et al., 2018). In 2020, the ACOG-SMFM proposed that for women who wish to evaluate their pregnancy for submicroscopic chromosomal change, prenatal diagnostic testing with CMA is recommended, however, no specific clinical indications for the application of CMA was explicitly proposed in this practice bulletin (Rose et al., 2020). Thus, for patients without fetal ultrasound abnormalities, there is no clear consensus on whether it is necessary to routinely recommend genetic tests other than karyotyping. On the other hand, a recent study (Hay et al., 2018) reported that for patients undergoing prenatal diagnosis with structurally normal fetuses, 2.5% of fetuses with clinically significant chromosomal abnormalities would be missed if only karyotyping had been performed. Meanwhile, the authors also acknowledged the two major barriers to the use of CMA for women with structurally normal fetuses, the VUS results appeared frequently (5.4% in this study) and the high costs. This result suggests that for cases without fetal ultrasound abnormality, novel, rapid genetic detection methods that are superior to conventional RAD approaches are needed to improve the detection efficiency of pCNV while circumventing the negative impact of performing whole-genome CNV tests.

The PNBoBs™ assay could be one such method. Compared to other rapid aneuploidy detection techniques such as Quantitative fluorescence PCR (QF-PCR) and Fluorescent *in situ* hybridizations (FISH), despite its slightly higher overall costs, the PNBoBs™ retain the advantages of rapidity and accuracy while expanding the target disease from common aneuploidies to frequent microdeletion syndromes. Over the last decade, several studies have confirmed that combining PNBoBs™ with karyotyping can effectively improve prenatal pCNV detection. However, none of these studies specified whether it should be applied to patients with different referral indications, or how to use the results to make clinical decisions (Grati et al., 2015; Fang et al., 2018; Huang et al., 2018; Li et al., 2018; Miao et al., 2019; Tao et al., 2019; Farra et al., 2020). With the clinical application of PNBoBs, different perspectives have emerged; some have suggested that it can replace traditional karyotyping (Piotrowski et al., 2012), arguing that PNBoBs™ can detect most clinical cases of pCNV and that it possesses advantages over traditional karyotyping. However, a more recent, alternative view is that PNBoBs™ should be replaced by genome-wide CNV analytical techniques, such as CNV-seq (Xu et al., 2020), regardless of the indication for prenatal diagnosis. These different views reflect the complexity of and lack of consensus on application criteria for PNBoBs™ in prenatal diagnosis.

Current guidelines recommend CMA as a first-tier test, but only when a fetal structural anomaly has been detected by ultrasound. However, with other indications for prenatal diagnosis, there are currently no sufficient evidence-based guidelines to support the diagnostic value of CMA. Several studies have reported the detection rate of the PNBoBs™ assay in patients with different indications for prenatal diagnosis, but their classification methods differed. Some classified all fetal ultrasound findings as ultrasound anomalies (Fang et al., 2018; Huang et al., 2018; Tao et al., 2019; Wang Y. et al., 2020), while others further divided findings into ultrasound structural malformations (FUSA) and soft-marker abnormalities (FUSMA) according to existing guidelines (Rosenfeld et al., 2014; Grati et al., 2015). We adopted the latter classification method for this study. Three cases of pCNV in the FUSA group that were missed by karyotyping were detected by PNBoBs™. Thus, the pCNV detection rate in our FUSA group [83% (10/12)] was much higher than that reported by Rosenfeld et al. [16.4% (108/660)] (Rosenfeld et al., 2014) and Grati et al. [18.7% (360/1922)] (Grati et al., 2015). This difference may be attributed to the small size of the FUSA group in our cohort. In the HR-NIPT group, 53% (10/19) of the cases were positive using either method. This finding was consistent with the rates reported by Huang et al. [50% (3/6)] (Huang et al., 2018)and Tao et al. [54% (20/43)] (Tao et al., 2019), indicating the efficacy of PNBoBs™ for rapid exclusion or confirmation of common fetal aneuploidies in pregnant women positive for NIPT. Of the five structurally normal cases of pCNV with normal karyotypes detected by PNBoBs™, three were in the HR-MSS group, with one case each in the FUSMA and AMA groups. These data suggest that PNBoBs™ is helpful for improving the detection rate of pathogenic variations in prenatal diagnosis for cases without abnormal structural features detectable by ultrasound.

In the present study, all 29 common aneuploidies and one case of translocation Down syndrome [46,XY,+21,der (21; 21) (q10; q10)] were quickly detected using PNBoBs™ within 48 h of sampling. Consistent with other studies (Tao et al., 2019; Xu et al., 2020), we found that PNBoBs™, at least for common aneuploidies, was highly concordant with karyotyping. Some have recommended that a positive RAD result, without karyotyping, can be directly used as a basis for medical decision-making in clinical management (Ogilvie et al., 2005; Comas et al., 2010). Our results also support this recommendation; however, it remains to be demonstrated that karyotyping helps rule out the possibility of translocation trisomies to provide accurate genetic counseling regarding recurrence risk. Moreover, 38 cases of balanced structural rearrangement, three cases of autosomal mosaicism, two cases of supernumerary marker chromosome, and two cases of unbalanced structural variation in our study were detected only by karyotyping. Among these, six cases showed pathogenic variations. These results indicate that the PNBoBs™ assay cannot completely replace karyotyping in prenatal diagnosis.

In a recent study comparing the detection performance of genome-wide copy-number analysis to PNBoBs™ in prenatal diagnosis (Xu et al., 2020), 10 cases of ambiguous results and two cases of sex-chromosome mosaicism were classified as false negatives by PNBoBs™. However, comparable types of karyotypes were accurately detected using PNBoBs™ in our study, which may be due to our improved data interpretation method for the PNBoBs™ assay (Jiang et al., 2021); If this hypothesis holds, then the rate of diagnostic agreement between CNV-seq and PNBoBs™ in Xu’s study would be 99.8% (1,873/1,876), indicating that the performance of PNBoBs™ may not be inferior to that of genome-wide copy number analysis, without distinguishing clinical indications. Moreover, unexpected findings and/or variants of unknown significance (VUS) identified by genome-wide CNV testing are still very challenging in terms of diagnostic assessment and genetic counseling (Durham et al., 2019). In Xu’s study, VUS accounted for 1.65% of the 1,876 cases assessed using CNV-seq (Xu et al., 2020). In contrast, PNBoBs™ provided explicit results, with clear genotype-phenotypic correlations (Vialard et al., 2011); thus, it is not likely to create ambiguity or confusion for genetic counseling. Furthermore, unlike PNBoBs™, none of the whole-genome CNV tests have regulatory approval for diagnostic use. Due to access restrictions, for prenatal diagnostic cases with indications for whole-genome CNV testing, most prenatal diagnosis centers in China must send samples to a third-party medical testing laboratory. The long-range transport of such samples inevitably increases the reporting turnaround time (approximately 3 weeks), increasing the risk of detection failure. Therefore, at present, we do not recommend whole-genome copy-number analysis for all patients undergoing invasive prenatal diagnosis. Nevertheless, because PNBoBs™ cannot accurately define the range of CNV, we recommend a validation test for CNV classified as incidental findings.

The types of samples tested with PNBoBs™ in previous studies included amniotic fluid (Gross et al., 2011; Vialard et al., 2012; García-Herrero et al., 2014; Fang et al., 2018; Huang et al., 2018; Li et al., 2018; Miao et al., 2019; Tao et al., 2019; Wang Y. et al., 2020; Farra et al., 2020; Xu et al., 2020), umbilical cord blood (Vialard et al., 2012; García-Herrero et al., 2014; Huang et al., 2018; Wang Y. et al., 2020; Farra et al., 2020), and chorionic villi (Huang et al., 2018; Wang Y. et al., 2020; Xu et al., 2020). We tested two types of samples: 2,364 amniotic fluid and four umbilical cord blood samples with specific features for fetal ultrasound abnormalities. The reason why we did not include chorionic villi samples (CVS) is that they carry a higher incidence of mosaicism owing to confined placental mosaicism (Wang et al., 1993). For CVS, if a positive result is obtained by PNBoBs™, further experimental verification is needed from an amniocentesis (Zhong et al., 2020), which will lead to increased medical expenses and a longer detection period. For umbilical cord blood, visual inspection is not suitable for assessing MCC; this will increase the risk of erroneous conclusions (Nagan et al., 2011). In contrast, although it is not always reliable, visual inspection is still considered a convenient way to assess MCC in amniotic fluid (Winsor et al., 1996). Therefore, to ensure the reliability and timeliness of the PNBoBs™ assay and leave enough time for follow-up pregnancy management, amniotic fluid collected in the second trimester of pregnancy is the ideal biological specimen for PNBoBs™ testing.

In conclusion, this study addressed the applicable populations, appropriate sample type, and clinical pathway for the PNBoBs™ assay in invasive prenatal diagnosis. We found that combining karyotyping with PNBoBs™ can improve both the diagnostic yield and efficiency of prenatal diagnosis and could be recommended in the second trimester in all patients without fetal structural anomalies who undergo invasive prenatal diagnosis, as a supplement to the parts not covered by the current guideline consensus, However, one limitation that needs to be noted is that the origins of all CNVs detected by PNBoBs™ could not be confirmed because of the refusal of some patients’ family members. Moreover, further investigations are required to better understand the phenotype-genotype correlations of microduplications in target regions with detailed clinical follow-up.

## Data Availability

The original contributions presented in the study are included in the article/Supplementary Material, further inquiries can be directed to the corresponding authors.
