# Supplementary material for: Clinical Utility of the Prenatal BACs-on-Beads™ Assay in Invasive Prenatal Diagnosis
Source: Front Genet. 2022 Jan 14;12:789625. doi: 10.3389/fgene.2021.789625 (PMC8795869; doi:10.3389/fgene.2021.789625)
Supplement: Supplementary file 1 [file Table1.DOCX]

Table s1 Details of pathogenic copy number variation cases detected by karyotyping and/or Prenatal Bobs™ assay

| Case No. | Clinical indication  for prenatal diagnosis | Karyotype | Chromosome or target MMS involved | PNBobs™  results |
| --- | --- | --- | --- | --- |
| 1 | HR-NIPT | 47,XX,+21 | Chr21 | Dup detected |
| 2 | HR-MSS | 47,XX,+21 | Chr21 | Dup detected |
| 3 | HR-NIPT | 47,XY,+21 | Chr21 | Dup detected |
| 4 | HR-MSS | 47,XY,+21 | Chr21 | Dup detected |
| 5 | HR-NIPT | 47,XX,+21 | Chr21 | Dup detected |
| 6 | HR-NIPT | 47,XX,+21 | Chr21 | Dup detected |
| 7 | AMA | 47,XY,+21 | Chr21 | Dup detected |
| 8 | FUSMA | 47,XX,+21 | Chr21 | Dup detected |
| 9 | HR-NIPT | 47,XY,+21 | Chr21 | Dup detected |
| 10 | HR-NIPT | 47,XY,+21 | Chr21 | Dup detected |
| 11 | HR-MSS | 47,XX,+21 | Chr21 | Dup detected |
| 12 | HR-MSS | 47,XY,+21 | Chr21 | Dup detected |
| 13 | FUSMA | 47,XX,+21 | Chr21 | Dup detected |
| 14 | APH | 46,XY,+21,der(21;21)(q10;q10) | Chr21 | Dup detected |
| 15 | FUSA | 45,XY,-21 | Chr21 | Del detected |
| 16 | FUSA | 47,XY,+18 | Chr18 | Dup detected |
| 17 | FUSA | 47,XY,+18 | Chr18 | Dup detected |
| 18 | HR-NIPT | 47,XX,+18 | Chr18 | Dup detected |
| 19 | FUSA | 47,XY,+13 | Chr13 | Dup detected |
| 20 | FUSA | 47,XY,+13 | Chr13 | Dup detected |
| 21 | HR-NIPT | 47,XX,+13 | Chr13 | Dup detected |
| 22 | HR-MSS | 47,XXY | ChrX | Dup detected |
| 23 | HR-NIPT | 47,XXY | ChrX | Dup detected |
| 24 | HR-MSS | 47,XXY | ChrX | Dup detected |
| 25 | FUSMA | 47,XXY | ChrX | Dup detected |
| 26 | HR-MSS | 47,XXY | ChrX | Dup detected |
| 27 | FUSMA | 47,XXX | ChrX | Dup detected |
| 28 | HR-MSS | 47,XYY | ChrY | Dup detected |
| 29 | HR-NIPT | 47,XYY | ChrY | Dup detected |
| 30 | FUSA | 45,X | ChrX | Del detected |
| 31 | HR-MSS | mos 45,X[63]/47,XXX[23] | ChrX | Del detected |
| 32 | AMA | mos 46,XY[45]/47,XXY[8] | ChrX | Dup detected |
| 33 | AMA | mos 45,X[37]/46,XX[20] | ChrX | Del detected |
| 34 | HR-MSS | mos 45,X[19]/46,XX[31] | ChrX | Del detected |
| 35 | HR-MSS | mos 45,X[6]/46,XY[69] | ChrY | Del detected |
| 36 | AMA | mos 45,X[8]/46,X,psu dic(Y)(q12)[52] | ChrY | Del detected |
| 37 | HR-MSS | mos 45,X[54]/46,XY[7] | ChrY | Del detected |
| 38 | FUSMA | mos 46,XX,t(3;7)(q24;q11.2)[14]/46,XX[36] | Chr3/7 | Undetected |
| 39 | HR-MSS | mos 46,XY,r(22)(p11.2q13)[21]/46,XY[83] | Chr22 | Undetected |
| 40 | FUSMA | 46,XY | PWS | Del detected |
| 41 | HR-MSS | 46,XY,del(5)(p15.3) | CDC | Del detected |
| 42 | HR-MSS | mos 48,XN,+5,+12[4]/46,XN[57] | Chr5/12 | Undetected |
| 43 | FUSA | 46,XY | MDS | Del detected |
| 44 | FUSA | 46,XY | DGS | Del detected |
| 45 | FUSA | 46,XY | DGS | Del detected |
| 46 | FUSA | 46,XY,del(4)(p16.1) | WHS | Del detected |
| 47 | HR-MSS | 46,XY,del(4)(p16.1) | WHS | Del detected |
| 48 | AMA | 46,XY | SMS | Del detected |
| 49 | HR-MSS | 46,XY | Xp22.31 | Del detected |
| 50 | HR-MSS | 46,XY | Xp22.31 | Del detected |
| 51 | HR-MSS | 46,XX | Xp22.31 | Del detected |
| 52 | HR-MSS | 46,XX,der(18)t(4;18)(q31.3;q22)mat | Chr4/18 | Undetected |
| 53 | HR-MSS | 46,XX,add(15)(q26.2)dn | Chr15 | Undetected |
| 54 | HR-MSS | 47,XY,+mar | Chr6 | Undetected |

Abbreviation: MMS: microdeletion/microduplication syndromes; PNBoBs™: prenatal bacterial artificial chromosome (BACs)-on beads; AMA, advanced maternal age; HR-MSS, high risk of maternal serological screening; HR-NIPT, high risk of noninvasive prenatal testing; FUSMA, fetal ultrasound soft markers abnormality; FUSA, fetal ultrasound structural abnormality; APH, adverse pregnancy history; PWS, Prader-Willi syndrome; MDS, Miller-Dieker syndrome; DGS, DiGeorge syndrome; WHS, Wolf-Hirschhorn syndrome; CDC, Cri du Chat syndrome; SMS, Smith-Magenis syndrome.
